# Hydrogen Absorption Reactions of Hydrogen Storage Alloy LaNi_5_ under High Pressure

**DOI:** 10.3390/molecules28031256

**Published:** 2023-01-27

**Authors:** Toyoto Sato, Hiroyuki Saitoh, Reina Utsumi, Junya Ito, Yuki Nakahira, Kazuki Obana, Shigeyuki Takagi, Shin-ichi Orimo

**Affiliations:** 1 Department of Engineering Science and Mechanics, College of Engineering, Shibaura Institute of Technology, Tokyo 135-8548, Japan; 2 Quantum Beam Science Research Directorate, National Institutes for Quantum Science and Technology, Sayo 679-5148, Japan; 3 Institute for Materials Research, Tohoku University, Sendai 980-8577, Japan; 4 Advanced Institute for Materials Research (WPI-AIMR), Tohoku University, Sendai 980-8577, Japan

**Keywords:** hydrogen storage material, high-pressure, synchrotron radiation X-ray diffraction

## Abstract

Hydrogen can be stored in the interstitial sites of the lattices of intermetallic compounds. To date, intermetallic compound LaNi_5_ or related LaNi_5_-based alloys are known to be practical hydrogen storage materials owing to their higher volumetric hydrogen densities, making them a compact hydrogen storage method and allowing stable reversible hydrogen absorption and desorption reactions to take place at room temperature below 1.0 MPa. By contrast, gravimetric hydrogen density is required for key improvements (e.g., gravimetric hydrogen density of LaNi_5_: 1.38 mass%). Although hydrogen storage materials have typically been evaluated for their hydrogen storage properties below 10 MPa, reactions between hydrogen and materials can be facilitated above 1 GPa because the chemical potential of hydrogen dramatically increases at a higher pressure. This indicates that high-pressure experiments above 1 GPa could clarify the latent hydrogen absorption reactions below 10 MPa and potentially explore new hydride phases. In this study, we investigated the hydrogen absorption reaction of LaNi_5_ above 1 GPa at room temperature to understand their potential hydrogen storage capacities. The high-pressure experiments on LaNi_5_ with and without an internal hydrogen source (BH_3_NH_3_) were performed using a multi-anvil-type high-pressure apparatus, and the reactions were observed using in situ synchrotron radiation X-ray diffraction with an energy dispersive method. The results showed that 2.07 mass% hydrogen was absorbed by LaNi_5_ at 6 GPa. Considering the unit cell volume expansion, the estimated hydrogen storage capacity could be 1.5 times higher than that obtained from hydrogen absorption reaction below 1.0 MPa at 303 K. Thus, 33% of the available interstitial sites in LaNi_5_ remained unoccupied by hydrogen atoms under conventional conditions. Although the hydrogen-absorbed LaNi_5_H_x_ (x < 9) was maintained below 573 K at 10 GPa, LaNi_5_H_x_ began decomposing into NiH, and the formation of a new phase was observed at 873 K and 10 GPa. The new phase was indexed to a hexagonal or trigonal unit cell with a ≈ 4.44 Å and c ≈ 8.44 Å. Further, the newly-formed phase was speculated to be a new hydride phase because the Bragg peak positions and unit cell parameters were inconsistent with those reported for the La-Ni intermetallic compounds and La-Ni hydride phases.

## 1. Introduction

The utilization of renewable energy is key to reducing the emission of greenhouse gases. Thus, a hydrogen-based society comprising hydrogen production by renewable energy, hydrogen storage, and hydrogen utilization is required for the effective utilization of renewable energy. This indicates that efficient and safe hydrogen storage methods are indispensable in a hydrogen-based society. Pressurized gaseous hydrogen, liquid hydrogen, and metal hydrides (hydrogen storage materials) are prime candidates for hydrogen storage methods [1,2,3,4,5]. Among them, hydrogen in molecular states (H_2_) is stored in the pressurized gaseous and liquid hydrogen, and hydrogen in atomic states (H) is stored in the hydrogen storage materials. Hydrogen storage capacity, an important factor in evaluating hydrogen storage methods, can be defined by its volumetric and gravimetric hydrogen densities. The total amount of hydrogen per volume is referred to as the volumetric hydrogen density, where a larger value indicates a more compact hydrogen storage method. The total amount of hydrogen per mass or weight is referred to as the gravimetric hydrogen density, where a larger value indicates a lighter hydrogen storage method. Notably, the volumetric hydrogen densities of pressurized hydrogen and liquid hydrogen cannot exceed 70 kgH_2_/m^3^, owing to the repulsion between hydrogen molecules. However, the volumetric hydrogen densities of most hydrogen storage materials can exceed 90 kgH_2_/m^3^ because hydrogen storage materials are stored hydrogen as atoms, which avoids repulsive interactions between the hydrogen molecules [1,2]. The high volumetric hydrogen densities of hydrogen storage materials are one of the advantages of the three methods. In addition, hydrogen storage materials exhibit reversible hydrogen absorption and desorption reactions under moderate pressure and temperature conditions (e.g., room temperature and below 1 MPa of hydrogen pressure). In the case of pressurized gaseous hydrogen, gaseous hydrogen is compressed under 70 MPa of hydrogen pressure for fuel-cell vehicles. Liquid hydrogen is obtained at 20–30 K. However, practically used hydrogen storage materials, such as LaNi_5_-based alloys, can undergo hydrogen absorption and desorption reactions at room temperature below 1 MPa of hydrogen pressure. Although hydrogen storage materials have disadvantages, such as low gravimetric hydrogen densities (e.g., gravimetric hydrogen density of LaNi_5_: 1.38 mass%), they are still considered efficient and safe hydrogen storage methods compared to gaseous or liquid hydrogen storage methods, owing to their high volumetric hydrogen densities and capability to undergo reversible hydrogen absorption and desorption reactions in moderate conditions. Therefore, many hydrogen storage materials have been reported [1,2,3,4,5].

The hydrogen absorption and desorption reactions of hydrogen storage materials involve the reaction of materials under hydrogen gas pressure. Hydrogen absorption reactions correspond to the formation of hydride phases, and hydrogen desorption reactions correspond to the decomposition of the hydride phases [1,2]. In hydrides, hydrogen basically exists in various states, including elemental hydrogen (hydrogen with charge neutral), ionic (protons (H^+^) and hydride ions (H^−^)), and covalently bonded hydrogen. For this reason, hydrogen storage materials are classified by their respective hydrogen states in their hydrides, of which there are three types: interstitial hydrides with elemental hydrogen; ionic hydrides with hydride ions; and complex hydrides with covalently bonded hydrogen [1,2,3,4,5,6,7,8,9,10,11,12]. In addition, porous materials adsorb hydrogen on their surfaces as molecular hydrogen (physisorption) [13,14,15].

Among the hydrides, interstitial hydrides are typical hydrogen storage materials, and some of them (e.g., LaNi_5_-based alloys) are practically used. The reasons are that ionic hydrides (e.g., MgH_2_) and complex hydrides (e.g., NaAlH_4_) can undergo hydrogen absorption and desorption reactions at relatively higher temperatures (typically several hundred degrees Celsius) and pressures (above several MPa of hydrogen gas pressures) because of thermodynamically stable phases, slow hydrogen absorption, and desorption reaction kinetics although their gravimetric hydrogen densities are higher than interstitial hydrides. Intermetallic compounds are often considered to form interstitial hydrides, which can also undergo stable hydrogen absorption and desorption reactions under moderate conditions, as mentioned above. During the hydrogen absorption reactions of intermetallic compounds, hydrogen atoms are located at the interstitial sites, which are typically octahedral and tetrahedral sites coordinated by six and four metal atoms in their lattices, respectively, while maintaining their atomic metal configurations [1,2,3,4,5]. The absorbed hydrogen atoms in the hydrides are released as hydrogen gas by controlling the pressure and/or temperature of the hydrogen gas.

**Figure 1:**
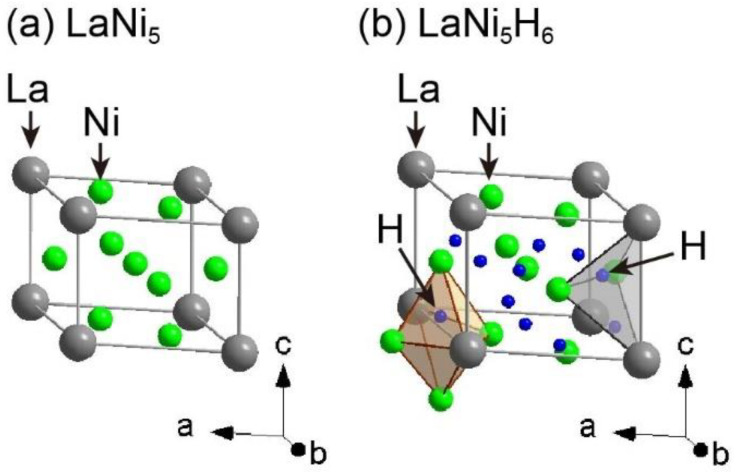
Crystal structures of (**a**) LaNi_5_ with a = 5.013 Å and c = 3.987 Å in the space group *P*6/*mmm* (No. 191) [21], and (**b**) LaNi_5_H_6_ with a = 5.410 Å and c = 4.293 Å in the space group *P*31*m* (No. 157) [18]. Gray, green, and blue spheres indicate La, Ni, and H atoms, respectively. The brown octahedron and gray tetrahedron denote H atomic sites at 3c and 6d, respectively.

Among hydrogen storage materials, LaNi_5_-based alloys of the AB_5_ alloy type (A and B are typically lanthanides with high hydrogen affinities and transition metals with low hydrogen affinities, respectively) are well-known as practical hydrogen storage materials because of their ability to undergo reversible hydrogen absorption and desorption reactions at room temperature and below 1 MPa of hydrogen gas pressure. Therefore, many studies have investigated the hydrogen storage properties of LaNi_5_-based alloys, including the hydrogen absorption and desorption reaction cycles, crystal structures, microstructures, and morphologies [5,16,17,18,19,20,21,22,23,24,25,26,27,28,29,30]. The crystal structures of LaNi_5_ before and after hydrogen absorption are shown in Figure 1 [18,21]. LaNi_5_ has a hexagonal structure with a = 5.013 Å and c = 3.987 Å in the space group *P*6/*mmm* (No. 191) [21]. Although crystal structures for the hydrogen-absorbed phase LaNi_5_H_6_ with different space groups and unit cell parameters have been reported, we refer to a trigonal unit cell with a = 5.410 Å and c = 4.293 Å in the space group *P*31*m* (No. 157) [18] in this paper. In the crystal structure of LaNi_5_H_6_, hydrogen atoms are located at octahedral sites coordinated by two La and four Ni atoms; the tetrahedral sites are coordinated by two La and two Ni atoms. In the octahedral and tetrahedral sites, the site occupancies of the hydrogen atoms are 96% at 3*c* (octahedral sites) and 52% at 6*d* (tetrahedral sites) in the space group *P*31*m* (No. 157), respectively. The gravimetric and volumetric hydrogen densities of LaNi_5_ are 1.38 mass% and 92 kgH_2_/m^3^, respectively. Despite the higher volumetric hydrogen density of LaNi_5_ compared to that of gaseous and liquid hydrogen (less than 70 kgH_2_/m^3^), it is necessary to improve the low gravimetric hydrogen density.

Although studies on hydrogen absorption and desorption reactions in hydrogen storage materials are typically conducted below 10 MPa, hydrogen absorption reactions can be facilitated above 1 GPa because of the drastic increase in the chemical potential of hydrogen [31]. To apply pressures above 1 GPa, multi-anvil-type high-pressure apparatus or diamond anvil cells have often been employed. In the multi-anvil-type high-pressure apparatus experiments, internal hydrogen sources, which supply hydrogen during the high-pressure experiments, are usually enclosed for the hydrogenation reaction (hydrogen absorption) because it is difficult to supply such high pressures using gaseous hydrogen. Hydrogen sources must release hydrogen above 1 GPa at the lowest possible temperature. In addition, the released hydrogen should not be absorbed by the hydrogen sources but by the sample instead. This indicates that the internal hydrogen source did not exhibit a reversible reaction between hydrogen desorption and absorption above 1 GPa. To date, BH_3_NH_3_ (hydrogen content: 19.6 mass%), a mixture of NaBH_4_ and Ca(OH)_2_ (hydrogen content: 6.3 mass%), AlH_3_ (hydrogen content: 10.1 mass%), and LiAlH_4_ (hydrogen content: 10.6 mass%) have often been used as the internal hydrogen sources. BH_3_NH_3_ has the highest hydrogen content and releases hydrogen at approximately 373–473 K at ambient pressure, and has the lowest hydrogen release temperature among the candidate internal hydrogen sources. Furthermore, hydrogen sources are generally separated by BN with a hexagonal crystal structure because the released hydrogen from the hydrogen sources can pass through the BN. A high-pressure experiment above 1 GPa enables the synthesis of new hydrides with the potential for hydrogen storage, which is otherwise difficult to synthesize under conventional conditions. Using a multi-anvil-type high-pressure apparatus, in which samples are enclosed with hydrogen sources, novel hydrides such as Mg_7_TMH_x_ (TM: transition metals in groups 4 and 5) and transition metal complex hydrides with alkali or alkaline-earth metals and Al_3_FeH_4_ have been successfully synthesized [32,33,34,35,36,37,38,39].

Furthermore, synchrotron X-ray and neutron diffraction are important characterization techniques for observing the hydrogen absorption reaction processes and elucidating their mechanisms under high pressure, as reported for hydride formation processes [37,38,39]. As mentioned above, many intermetallic compounds classified as interstitial hydrides undergo reversible hydrogen absorption and desorption reactions at room temperature and above ambient pressure. This indicates that the hydrogen-absorbed phases (hydride phases) must be observed under hydrogen pressure because the absorbed hydrogen atoms are released from the intermetallic compounds at ambient pressure. To date, many new hydrides with high hydrogen contents have been reported. Most are ionic or complex hydrides, such as hydrogen with intermediate hydrogen states, which can be recovered at ambient pressure because they are thermodynamically stable hydrides. To our knowledge, a typical hydrogen storage material, LaNi_5_, has not yet been investigated for hydrogen absorption reactions above 1 GPa, although a TiFe with reversible hydrogen absorption and desorption reactions below 10 GPa has been reported [40].

In this study, we conducted hydrogen absorption reactions on LaNi_5_, a typical hydrogen storage material, above 1 GPa at room temperature, focusing on investigating its potential hydrogen storage capacity.

## 2. Results and Discussion

### 2.1. Hydrogen Absorption and Desorption Reactions below 1 MPa

**Figure 2:**
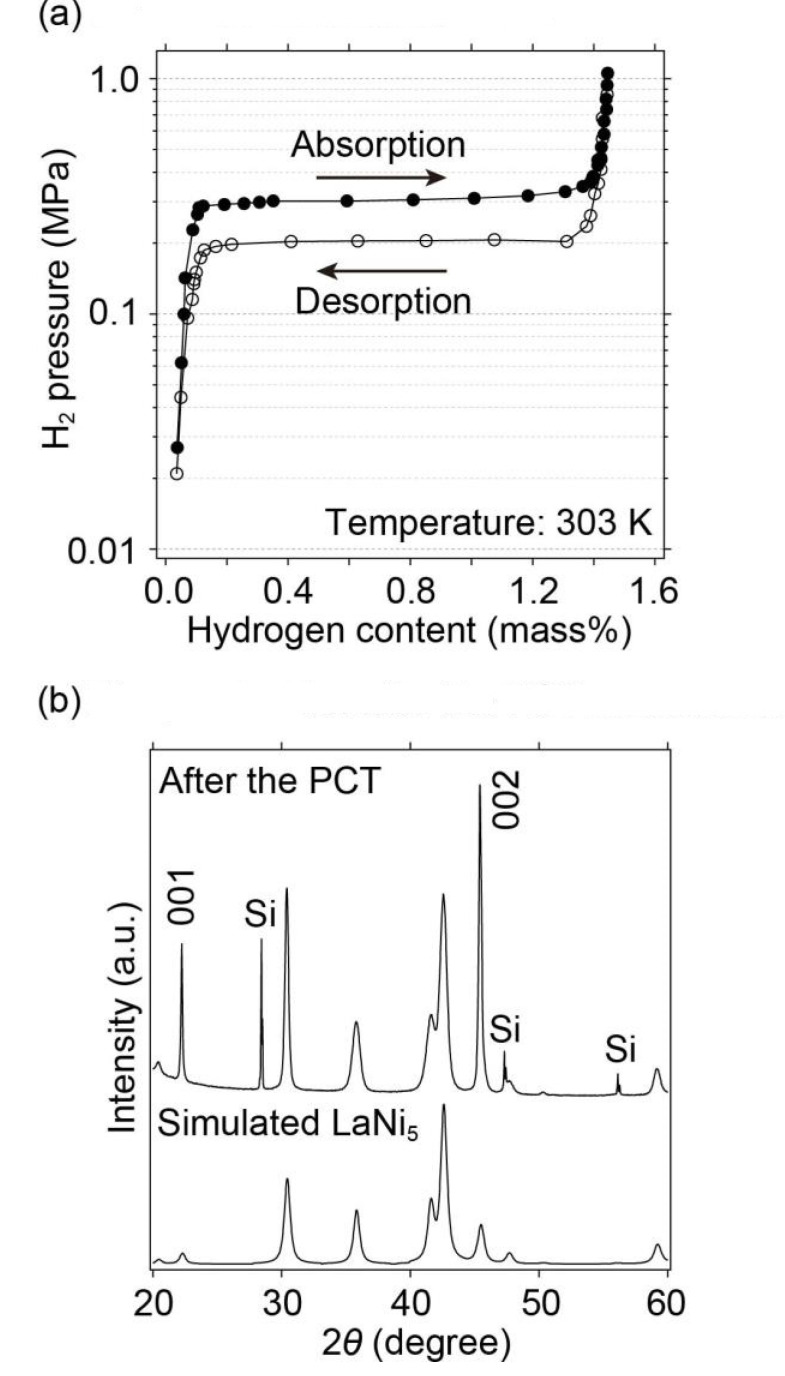
(**a**) PCT curves of LaNi_5_ measured at 303 K, and (**b**) X-ray diffraction pattern after the PCT measurements with simulated X-ray diffraction pattern of LaNi_5_. In the X-ray diffraction pattern after the PCT measurement, strong intensities of (001) and (002) reflections originated into a preferred orientation of (00*l*) reflection. Si is added as an internal standard in the X-ray diffraction pattern after the PCT measurement.

In this study, the hydrogen absorption and desorption of LaNi_5_ were measured using a Pressure Composition Temperature (PCT) apparatus at 303 K below 1 MPa (Figure 2). The observed hydrogen storage capacity (1.40 mass%) and equilibrium hydrogen absorption and desorption pressures (0.3 and 0.2 MPa, respectively), including hysteresis, which would originate from strain in atomic arrangements, were consistent with those reported by Liang et al. in [24]. Then, the samples were characterized by a conventional X-ray diffraction diffractometer, as shown in Figure 2. Although the preferred orientation (001) at 2θ ≈ 22.2°, and (002) at 2θ ≈ 45.4°, and Bragg peak broadening were observed, the Bragg peak positions and unit cell parameters, hexagonal unit cells with a = 5.013(2) Å, and c = 3.988(1) Å obtained by the indexing program TREOR97 [41], were consistent with the simulated X-ray diffraction pattern of LaNi_5_ with hexagonal unit cells (a = 5.013 Å, and c = 3.987 Å) [21]. This strain was observed by broadening the Bragg peaks in the X-ray diffraction pattern after the hydrogen absorption and desorption reactions, as reported in [22,24,25]. Therefore, this sample was used to observe the hydrogen absorption reactions of LaNi_5_ at pressures above 1 GPa, which will be discussed in the following section.

### 2.2. Hydrogen Absorption Reactions at 1.6 GPa and 473 K

**Figure 3:**
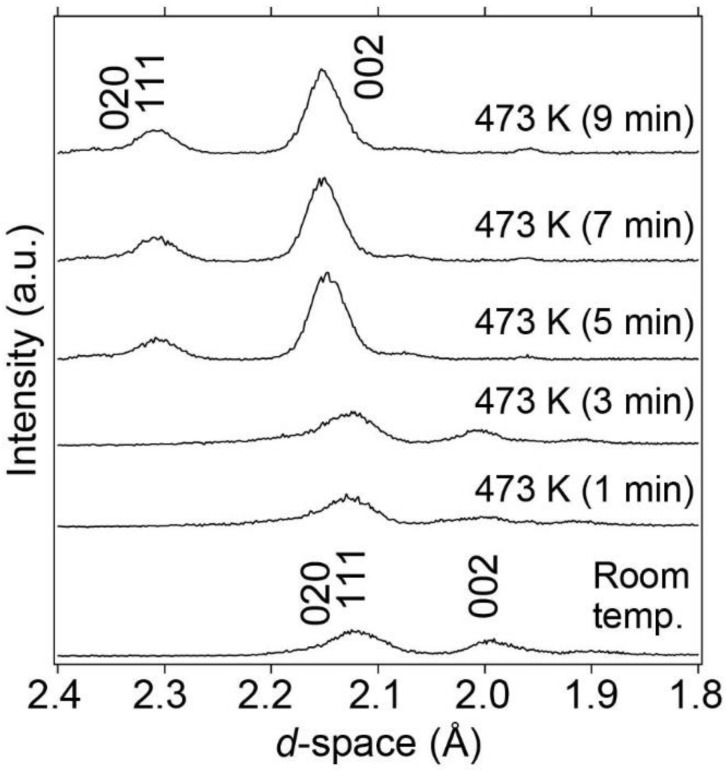
Synchrotron radiation X-ray diffraction patterns of LaNi_5_ at 473 K and 1.6 GPa focusing on Bragg peaks of the (020), (111), and (002) reflections.

LaNi_5_ with an internal hydrogen source BH_3_NH_3_ was compressed to 1.6 GPa at room temperature and subsequently heated to 473 K at a heating rate of 100 K/min to release hydrogen. During heating, synchrotron radiation X-ray diffraction patterns were collected every 1 min using the energy-dispersive method, as shown in Figure 3. After 5 min of reaction at 473 K and 1.6 GPa, the Bragg peaks shifted to larger *d*-spacings. This indicates that BH_3_NH_3_ released hydrogen, which was then absorbed by LaNi_5_ without yielding byproducts from the reaction between LaNi_5_ and BN capsules. The amount of absorbed hydrogen is estimated to be approximately 1.70 mass%, corresponding to the formation of LaNi_5_H_7.3_, considering the unit cell volume expansion. The details of the hydrogen content considered in this study are discussed later. After the hydrogen absorption reaction was completed, the temperature was lowered to room temperature while maintaining the pressure at 1.6 GPa to observe the hydrogen absorption reaction at room temperature with increasing pressure.

### 2.3. Hydrogen Absorption Reactions in the Range of 1–10 GPa

**Figure 4:**
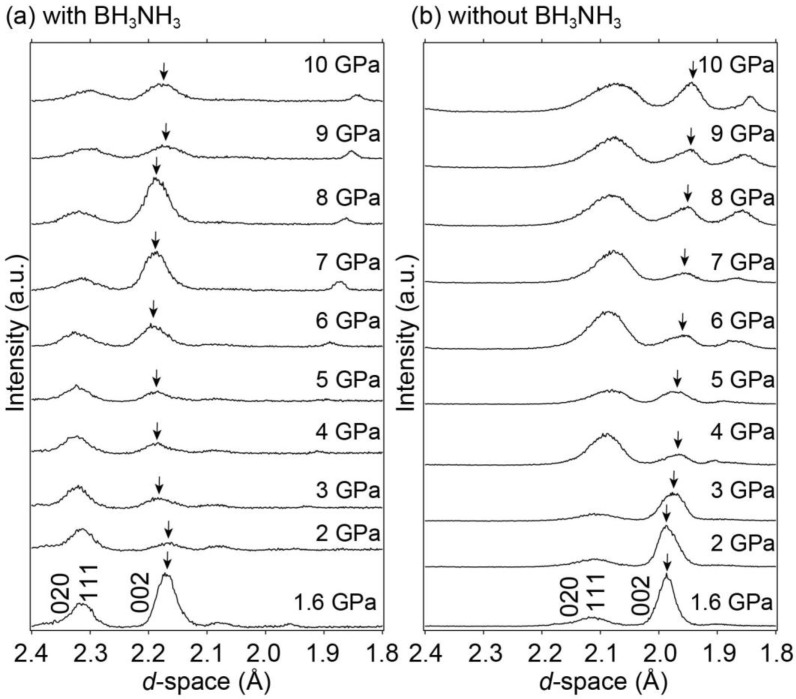
Synchrotron radiation X-ray diffraction patterns of LaNi_5_ (**a**) with and (**b**) without the internal hydrogen source BH_3_NH_3_ at room temperature and 1.6–10 GPa focusing on Bragg peaks of the (020), (111), and (002) reflections. Arrows indicate the Bragg peak of (002) at each pressure.

**Figure 5:**
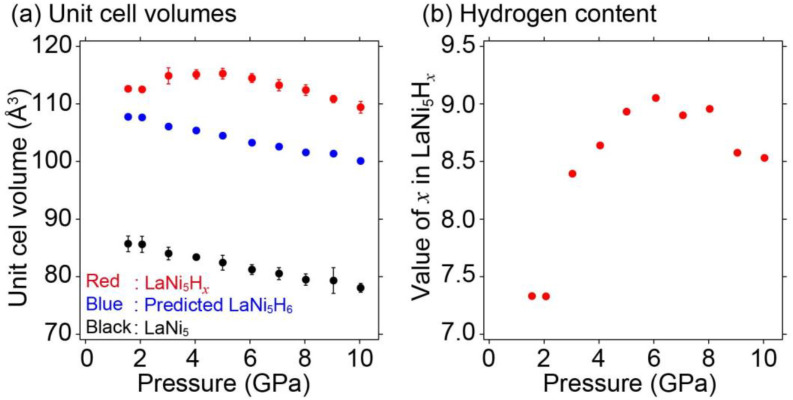
(**a**) Changes in the unit cell volume of LaNi_5_H_x_ (LaNi_5_ with BH_3_NH_3_) predicted LaNi_5_H_6_ and LaNi_5_ without BH_3_NH_3_, and (**b**) estimated hydrogen content in LaNi_5_H_x_ as a function of pressure.

Figure 4 shows the synchrotron radiation X-ray diffraction patterns of LaNi_5_ with and without BH_3_NH_3_ in the 1.6–10 GPa range at room temperature. High-pressure experiments with and without BH_3_NH_3_ were performed to observe the hydrogen absorption reaction of LaNi_5_ and study the unit cell volume expansion of pure LaNi_5_ with increasing pressure. Although it is difficult to evaluate Bragg peak intensities and widths due to data collection of the X-ray diffraction by the energy-dispersive method and sample positions, the Bragg peak positions of LaNi_5_ without BH_3_NIH_3_ shifted to smaller *d*-spacings with increasing pressure, corresponding to the decrease in the unit cell volume as higher pressure was applied. By contrast, the Bragg peak positions of LaNi_5_ with BH_3_NH_3_ either remained constant or shifted to larger *d*-spacing despite increasing pressure (1.6 to 6 GPa). This indicated that LaNi_5_ absorbed hydrogen during compression. Using the X-ray diffraction pattern fitting program, PDindexer [42], the experimentally obtained synchrotron radiation X-ray diffraction patterns were fitted to that of LaNi_5_H_6_ with a hexagonal structure in the space group *P*31*m* (No. 157) [18], and unit cell volumes at each pressure were estimated based on the fitting results. Figure 5 shows the pressure dependence of the unit cell volume. The unit cell volumes of LaNi_5_H*_x_* (LaNi_5_ with BH_3_NH_3_), predicted LaNi_5_H_6_, and LaNi_5_ (LaNi_5_ without BH_3_NH_3_) were plotted as a function of pressure. The unit cell volume of the predicted LaNi_5_H_6_ was estimated using the unit cell volume of LaNi_5_ (black data points in Figure 5) and the volume of the hydrogen contribution to LaNi_5_H_6_. The volume of the hydrogen contribution (22.05 Å^3^/f.u.) was calculated by subtracting the unit cell volume of LaNi_5_ (87.76 Å^3^/f.u. [21]) from that of LaNi_5_H_6_ (108.81 Å^3^/f.u. [18]). As shown in Figure 5, gaps were observed in the unit cell volumes of LaNi_5_H_x_ and the predicted LaNi_5_H_6_. These gaps were proposed to hold extra hydrogen in LaNi_5_H_6_ for the high-pressure experiment. Because the volume of hydrogen contribution was 22.05 Å^3^/f.u. in LaNi_5_H_6_, each hydrogen atomic contribution was estimated to be 3.68 Å^3^/f.u. Therefore, the maximum hydrogen storage capacity reaches 2.07 mass% (LaNi_5_H_9_) at 6 GPa. Notably, a similar intermetallic compound, TiFe, with reversible hydrogen absorption and desorption reactions below 10 GPa, also showed the same hydrogen content under normal conditions and higher pressures [40].

**Figure 6:**
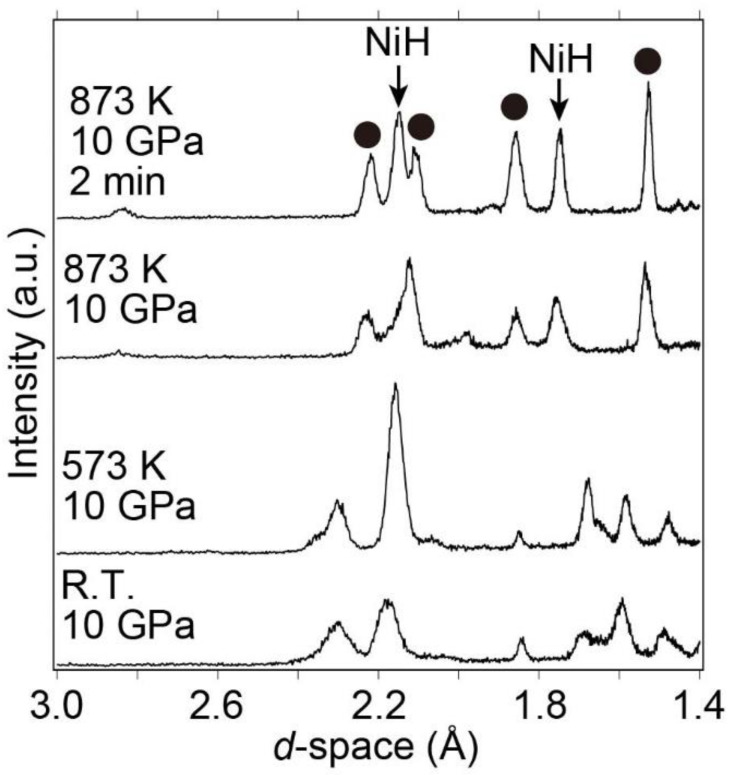
Synchrotron radiation X-ray diffraction patterns from room temperature to 873 K at 10 GPa. The Bragg peaks at 873 K and 10 GPa (2 min) are identified as NiH and a new phase with a hexagonal unit cell (closed circles).

Subsequently, the sample was heated from room temperature to 873 K at a pressure of 10 GPa to further investigate hydrogen absorption and decomposition (into La hydride (LaH_x_) and Ni) reactions at higher temperatures. X-ray diffraction patterns were simultaneously collected during heating. Bragg peaks corresponding to NiH and unknown Bragg peaks were observed at 873 K and 10 GPa although the hydrogen-absorbed LaNi_5_H_x_ (x < 9) was maintained below 573 K at 10 GPa (Figure 6). Using the indexing program TREOR97 [41], the unknown Bragg peaks were indexed to a hexagonal or trigonal unit cell with a ≈ 4.44 Å and c ≈ 8.44 Å. The new phase with the hexagonal or trigonal unit cell was speculated to be a new hydride phase because the Bragg peak positions and the phase with the hexagonal or trigonal unit cell did not match those of the La-Ni hydrides and La-Ni intermetallic compounds. Because the new phase could be recovered at an ambient pressure, it might not be classified as an interstitial hydride. The details of the new phase, including its atomic positions and hydrogen content, are currently being investigated.

Thus, the high-pressure experiments on LaNi_5_ suggest that its maximum hydrogen storage capacity of LaNi_5_ could be 2.07 mass% at 6 GPa, indicating that 33% of the available interstitial sites in LaNi_5_ remain unoccupied under practical operating conditions. Although hydrogen atoms in LaNi_5_ occupy octahedral sites coordinated by two La and four Ni atoms and tetrahedral sites coordinated by two La and two Ni atoms (see Figure 1), a small number of hydrogen atoms in LaNi_5_ with Al (LaNi_4.5_Al_0.5_) occupied other tetrahedral sites, coordinated by one La and three Ni atoms and by four Ni atoms, as reported by atomic pair distribution function analysis [30]. Therefore, it can be speculated that the hydrogen storage capacities might improve if more hydrogen atoms could occupy the respective tetrahedral sites coordinated by one La, three Ni atoms, and four Ni atoms. To increase the hydrogen atomic occupancies at the tetrahedral sites, replacing multiple metal atoms, which have a larger radius and higher affinity for hydrogen than Ni atoms, with Ni atomic sites might lead to increased amounts of hydrogen atoms in such tetrahedral sites.

## 3. Materials and Methods

Pieces of LaNi_5_ (Japan Metals & Chemicals Co., Ltd., Tokyo, Japan) were used in this study. The hydrogen storage properties of LaNi_5_ at 303 K and below 1 MPa of hydrogen gas pressure were confirmed by PCT measurements (Japan Metals & Chemicals Co., Ltd.). Before the PCT measurement, five cycles of hydrogen absorption and desorption reactions on LaNi_5_ were performed at room temperature under 1 MPa of hydrogen gas pressure and subsequently under vacuum for activation after heat treatment at 623 K for 3 h. After the PCT measurements, the samples were characterized by powder X-ray diffraction, which was conducted on a Rigaku SmartLab X-ray diffractometer (Tokyo, Japan) using Cu Kα radiation (λ = 1.540593 Å for Kα_1_ and λ = 1.544414 Å for Kα_2_). The samples were handled in an Ar-gas-filled glovebox with an O_2_ content < 1 ppm to prevent (hydro)oxidation.

The hydrogen absorption reactions of LaNi_5_ at room temperature and above 1 GPa were observed using a multi-anvil-type high-pressure apparatus combined with synchrotron radiation X-ray diffraction at the BL14B1 beamline of SPring-8 in Japan. Before the high-pressure experiment, LaNi_5_ was activated by heat treatment at 623 K for 3 h, and hydrogen absorption and desorption reactions as the same processes with the PCT measurement at 303 K, as mentioned above. Activated LaNi_5_ was placed in a BN capsule enclosed by an internal hydrogen source, BH_3_NH_3_ (Sigma-Aldrich, 97%, St. Louis, MO, USA). The weight ratio of LaNi_5_ to BH_3_NH_3_ was approximately 3:2. The sample and the internal hydrogen source were enclosed in a NaCl hydrogen-sealing capsule. The sample cell used in this study was based on that used by Saitoh et al. [38]. The hydrogen release reactions of BH_3_NH_3_ above 1 GPa have been described in [43,44]. Synchrotron radiation X-ray diffraction patterns were collected during high-pressure experiments using energy-dispersive X-ray diffraction. In high-pressure experiments, diffracted X-rays were obtained using a Ge solid-state detector mounted on a goniometer. The *d*-space of the lattice was obtained using Bragg’s law:

(1) $$d=\frac{hc}{2E\sin\theta }$$

where:

*h*: Planck constant;

*c*: Speed of light;

*E*: Energies of the diffracted X-ray;

*θ*: 3° (diffraction angle 2θ was fixed at 6° in this experiment).

High-pressure experiments on LaNi_5_ without an internal hydrogen source were also performed using the same procedure to compare the hydrogen absorption reactions. For these cases, BN was used instead of the internal hydrogen source.

In addition, the Bragg peaks in the experimentally obtained synchrotron radiation X-ray diffraction patterns were indexed using TREOR97 [41] and identified using PDindexer [42].

## 4. Conclusions

In this study, we investigated the hydrogen absorption and desorption reactions of LaNi_5_ below 1 MP at 303 K and above 1 GPa at room temperature to determine its potential hydrogen storage capacities. High pressures above 1 GPa were induced using a multi-anvil-type high-pressure apparatus, and the hydrogen absorption reactions of LaNi_5_ above 1 GPa were observed using in situ synchrotron radiation X-ray diffraction with an energy dispersive method at the BL14B1 beamline of SPring-8 in Japan. Although LaNi_5_ absorbed up to 1.40 mass% hydrogen, of which LaNi_5_H_6_ was formed below 1 MPa at 303 K, the results suggested that the hydrogen storage capacity increased with increasing pressure up to 6 GPa. Considering the unit cell volumes of hydrogen-absorbed LaNi_5_ phase LaNi_5_H_x_ (LaNi_5_ with an internal hydrogen source), predicted LaNi_5_H_6_ and LaNi_5_ (without an internal hydrogen source). The final amount of LaNi_5_ reached 2.07 mass%, corresponding to the formation of LaNi_5_H_9_ at 6 GPa and room temperature. This indicated that approximately 33% of the available interstitial sites in LaNi_5_ remained unoccupied by hydrogen atoms under practical operating conditions. The locations of the unoccupied hydrogen atomic sites were speculated to be in tetrahedral sites coordinated by one La, three Ni atoms, and four Ni atoms because small amounts of hydrogen could be located at the tetrahedral sites when a part of Ni was replaced with Al. To increase the hydrogen atomic occupancies at the tetrahedral sites, replacing multiple metal atoms, which have a larger radius and higher affinity for hydrogen than Ni atoms, with Ni atomic sites might lead to increased amounts of hydrogen atoms in unoccupied hydrogen atomic sites under normal conditions.

While increasing the temperature from room temperature to 873 K at 10 GPa, the hydrogen-absorbed phase LaNi_5_H_x_ (x < 9) decomposed to form NiH and a new phase at 873 K. The new phase was indexed to a hexagonal or trigonal unit cell with a ≈ 4.44 Å and c ≈ 8.44 Å. It was speculated to be a new hydride phase because its Bragg peak positions were inconsistent with La-Ni Intermetallic compounds and La-Ni hydrides.

## Data Availability

Not applicable.

## Sample Availability

Samples of the compounds are available from the authors.
